# Spinocerebellar ataxia 17: full phenotype in a 41 CAG/CAA repeats carrier

**DOI:** 10.1186/s40673-018-0086-x

**Published:** 2018-03-14

**Authors:** Paola Origone, Fabio Gotta, Merit Lamp, Lucia Trevisan, Alessandro Geroldi, Davide Massucco, Matteo Grazzini, Federico Massa, Flavia Ticconi, Matteo Bauckneht, Roberta Marchese, Giovanni Abbruzzese, Emilia Bellone, Paola Mandich

**Affiliations:** 1 Department of Neuroscience, Rehabilitation, Ophthalmology, Genetics and Maternal Child Health, University of Genova, c/o DIMI Viale Benedetto XV, 6 – 16132 Genova, Italy; 2 Medical Genetic Unit, Ospedale Policlinico San Martino, Genova, Italy; 3 Neurology Unit, Ospedale Antero Micone, Genova, Italy; 4 Clinical Neurology Unit, Ospedale Policlinico San Martino, Genova, Italy; 5 Department of Health Sciences, University of Genova, Genova, Italy; 6 Nuclear Medicine Unit, Ospedale Policlinico San Martino, Genova, Italy

**Keywords:** Spinocerebellar ataxia, *TBP* gene, SCA17, Incomplete penetrance

## Abstract

**Background:**

Spinocerebellar ataxia 17 (SCA17) is one of the most heterogeneous forms of autosomal dominant cerebellar ataxias with a large clinical spectrum which can mimic other movement disorders such as Huntington disease (HD), dystonia and parkinsonism. SCA17 is caused by an expansion of CAG/CAA repeat in the Tata binding protein (*TBP*) gene. Normal alleles contain 25 to 40 CAG/CAA repeats, alleles with 50 or greater CAG/CAA repeats are pathological with full penetrance. Alleles with 43 to 49 CAG/CAA repeats were also reported and their penetrance is estimated between 50 and 80%. Recently few symptomatic individuals having 41 and 42 repeats were reported but it is still unclear whether CAG/CAA repeats of 41 or 42 are low penetrance disease-causing alleles. Thus, phenotypic variability like the disease course in subject with SCA17 locus restricted expansions remains to be fully understood.

**Case presentation:**

The patients was a 63-year-old woman who, at 54 years, showed personality changes and increased frequency of falls. At 55 years of age neuropsychological tests showed executive attention and visuospatial deficit. At the age of 59 the patient developed dysarthria and a progressive cognitive deficit. The neurological examination showed moderate gait ataxia, dysdiadochokinesia and dysmetria, dysphagia, dysarthria and abnormal saccadic pursuit, severe axial asynergy during postural changes, choreiform dyskinesias. Molecular analysis of the *TBP* gene demonstrated an allele with 41 repeat suggesting that 41 CAG/CCG *TBP* repeats could be an allele associated with the full clinical spectrum of SCA17.

**Conclusions:**

The described case with the other similar cases described in the literature suggests that 41 CAG/CAA trinucleotides should be considered as critical threshold in SCA17. We suggest that SCA17 diagnosis should be suspected in patients presenting with movement disorders associated with other neurodegenerative signs and symptoms.

## Background

Spinocerebellar ataxia 17 (SCA17) is one of the most heterogeneous forms of autosomal dominant cerebellar ataxias with a large clinical spectrum which can mimic other movement disorders such as Huntington disease, dystonia and parkinsonism [1, 2]. In addition to gait and limb ataxia and dysarthria, the clinical picture may include parkinsonism, choreic movements, dystonia, cognitive and psychiatric symptoms. Furthermore, epilepsy and extrapyramidal signs may be also present [3–5].

Brain magnetic resonance imaging (MRI) usually shows marked cerebellar atrophy with milder cerebral atrophy and relative brainstem sparing.

SCA17 is caused by an expansion of CAG/CAA repeat in the Tata binding protein (*TBP*) gene [6]. The CAG/CAA repeat expansion shows intergenerational stability that may be due to the presence of the CAA interruption [1, 3, 7]. Normal alleles contain 25 to 40 CAG/CAA repeats while 50 or greater CAG/CAA repeats are alleles with full penetrance [8]. Alleles with 43 to 49 CAG/CAA repeats were also reported and their penetrance is estimated between 50 and 80%. It is still unclear whether CAG/CAA repeats of 41 or 42 are low penetrance disease-causing alleles, nevertheless few symptomatic individuals having 41 and 42 repeats were reported recently [9–11].

Herein we report a patient carrying 41 CAG/CAA repeats presenting with the SCA17 classical features.

## Case presentation 

The proband was a 63-year-old woman who suffered of a severe depressive episode at 46 years of age, then she slowly developed gait ataxia with falls and dysfunction of fine finger movements. She is the second child of unrelated Italian parents. The father died at 82 years of age with cognitive deterioration starting at 70; the mother is 87 years old and healthy. The patient has three healthy siblings and a son (34 years old) who is reported to suffer of epilepsy since his childhood and showing a wide-based gait in the last two years.

**Fig. 1:**
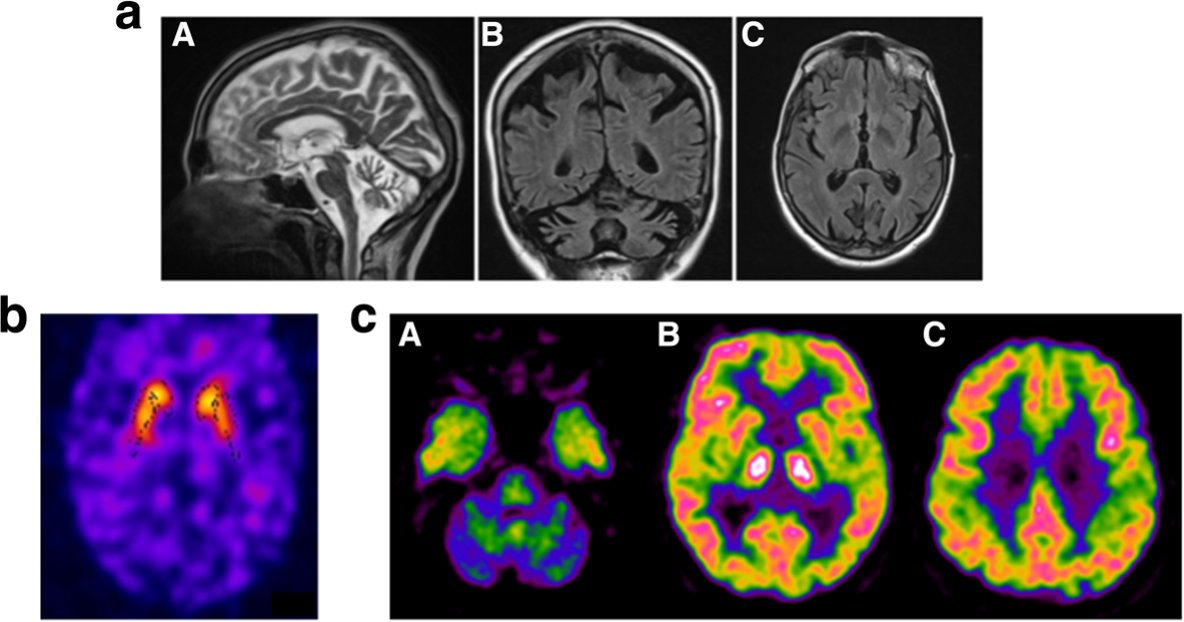
**a** Sagittal brain MRI (T2-weighted) disclosing gross and diffuse atrophy involving the cerebellum (A). Coronal brain MRI (T1-weighted) showing symmetrical atrophy of cerebellum and parietal cortex (B). Axial brain MRI (FLAIR) showing preservation of basal ganglia (C). **b** DAT-SPECT with I-123 ioflupane showed globally normal standardized binding ratios with only mild reduction of the putamen-to-caudate ratio in the left hemisphere (0.64). Semi quantification was achieved through the Basal ganglia software V2 (Calvini et al., [15]). **c** 18F- FDG PET showed severely decreased uptake in both cerebellar hemispheres (A) and moderate decreased uptake in the two putamen, mainly in the left hemisphere (B). No cortical uptake deficit was found (B, C). Hot colors (white, red, yellow) report higher uptake than cold colors (green, blue)

When she was 54, the patient’s relatives noticed personality changes (aggressiveness, hyperphagia, inappropriate shopping) and increased frequency of falls. Brain magnetic resonance imaging (MRI) showed bilateral and symmetric moderate atrophy of frontal and parietal cortex and clear atrophy of both cerebellum hemispheres and vermis. At that time a tentative diagnosis of olivo-ponto-cerebellar atrophy was made in another hospital. At 55 years of age neuropsychological tests (including Stroop color-word, Wisconsin Card Sorting, Rey auditory verbal learning, Clock drawing, Digit symbol, and Trail Making A and B tests) showed executive attention and visuospatial deficit. The mini-mental state examination (MMSE) score was 29/30. At the age of 59 the patient developed dysarthria. When she was 61 she suffered from generalized tonic-clonic seizures but the interictal electroencephalogram (EEG) was normal. There was progressive cognitive worsening and at the age of 62 the neuropsychological assessment showed deficit in verbal memory, language and attention, and the MMSE scored 15/30. The neurological examination showed moderate gait ataxia, dysdiadochokinesia and dysmetria, dysphagia, dysarthria and abnormal saccadic pursuit, severe axial asynergy during postural changes, choreiform dyskinesias involving upper limbs, face, and tongue. She had no muscle weakness or sensory deficits, deep tendon reflexes were normal and pyramidal signs were absent. At the time of the last examination when the patient was 63, she required assistance for everyday activities. Brain MRI showed advanced cerebellar atrophy, involving both hemispheres and vermis, with brainstem sparing (Fig. 1a). Brain single photon emission computed tomography (SPECT) using the Dopamine Transporter (DAT) ligand I-123 Ioflupane (DAT-SPECT) showed mild reduced uptake in left posterior putamen but was otherwise normal (Fig. 1b). F-18 Fluorodeoxyglucose positron emission tomography (FDG-PET) showed severely decreased glucose metabolism of both cerebellar hemispheres and putamen (Fig. 1c).

### Molecular analysis methods

The genetic analysis for *HD, DRPLA* and *TBP* was performed on genomic DNA extracted from peripheral blood sample by standard methods. The polymerase chain reaction (PCR) was performed by using the published primers [6] and PCR products were separated by capillary electrophoresis ABIPrism 3130XL Genetic Analyzer. The size of each allele was established by comparison with control DNAs previously determined by direct sequencing.

## Results

Routine laboratory investigations, including serum analysis of copper, ceruloplasmine, thyroxine and blood count, including test for acantocytosis were normal. Other causes of choreic syndromes (vascular/infectious, vitamin deficiencies, and metabolic disorders) were excluded. Molecular genetic testing for Huntington’s disease, dentatorubral-pallido-luysian atrophy, SCA1, SCA2, SCA3, SCA6, SCA7 were previously excluded in another laboratory. Informed consent for genetic testing was signed by the patient and by her sister (legal tutor).

Genetic analysis of the *TBP* showed two alleles of 36 and 41 CAG/CAA repeats. The analysis of *TBP* gene was repeated with matching results in one more laboratory, in blind.

## Discussion

Few SCA17 patients having 41 CAG/CCG have been reported so far, Nanda et al. [9] reported a patient with a prominent cerebellar involvement and a mild cognitive impairment with onset at age 50 years. Doherty et al. [12] reported a patient presenting at 59 years with late onset ataxia mimicking multiple system atrophy type C (MSA-C). Park et al. [13] described a patient presenting with parkinsonism and chorea with onset at age 61 years in whom the PET scan performed eight years after onset showed marked decrease of dopamine transporter (DAT) density in the bilateral putamen. Alibardi et al. [11] reported a patient with psychiatric onset and late chorea without cerebellar and pyramidal involvement.

As in our patient, in all these cases the diagnosis of SCA17 took some years and was performed only after excluding other acquired and genetic causes of movement disorders.

The wide clinical spectrum of SCA17 is challenging for diagnosis. In these patients, in spite of the same number of CAG/CCG which they carried, both the type of symptoms and signs at onset and the disease course are different, as shown in Table 1.

**Table 1:** Comparisons of clinical findings among SCA17 cases with 41 CAG/CCG repeats

Age at onset	Signs and symptoms at onset	Psychiatric symptoms	MRI	PET	Age^a^	Neurological examination	Reference
54	Personality changes increased frequency of falls	Depressive status, personality changes	Advanced cerebellar atrophy	Severely decreased glucose metabolism of both cerebellar hemispheres and putamen	63	Gait ataxia, dysdiadochokinesia and dysmetria, dysphagia, dysarthria and abnormal saccadic pursuit, severe axial asynergy during postural changes, choreiform dyskinesias	Present study
50	Gait difficulties increased frequency of falls	No	Cerebellar atrophy	na	75	Dysarthric speech, limb ataxia, gait ataxia, and brisk reflexes	Nanda et al., [9]
60	Involuntary movements of the limbs	Depressive status	na	na	63	Generalized choreic movements	Alibardi et al, [11]
59	Dysarthria and gait ataxia	No	Cerebellar atrophy	na	na	Pure cerebellar syndrome	Doherty et al., [12]
61	Hand tremor and progressive gait disturbance	No	No cerebellar atrophy	Markedly decreased DAT density in the bilateral putamen with anteroposterior gradient	64	Flexed posture and a short-step gait with a decreased bilateral arm swing resting-type hand tremor and bradykinesia. Choreic mixed with stereotypic and dystonic movements in perioral area and both hands	Park et al., [13]

^a^At neurological examination; na: not available

Moreover, probably because of the low number of *TBP* repeats associated with incomplete penetrance, the family history is often negative and therefore useless. In the present case, a near-normal DAT SPECT with a full-blown clinical picture virtually rules out both Parkinson’s disease and MSA, although some degree of reduced DAT uptake has been reported in SCA 17 patients as well [14].

There have been few case reports of patients with a typical phenotype and 41 repeats, suggesting a moderate disease course [9–11, 13], which is not the case with our patient. Despite our patient carried 41 repeat expansion, she developed a typical phenotype with chorea and early-onset dementia. Thus, phenotypic variability like the disease course in subject with SCA17 locus restricted expansions remains to be fully understood.

## Conclusions

We believe that 41 CAG/CCG *TBP* repeats is an allele which can be associated with the full clinical spectrum of SCA17, although with incomplete penetrance. The described case with the other similar cases described in the literature suggests that 41 CAG/CAA trinucleotides should be considered as critical threshold in SCA17. These findings support the idea that a degree of CAG/CAA repeats correlate with penetrance but not the phenotype variability. Therefore, due to pleiotropic effects of *TBP* gene, we suggest that SCA17 diagnosis should be suspected in patients presenting with movement disorders associated with other neurodegenerative signs and symptoms.

## Abbreviations

DAT: Dopamine transporter
DRPLA: Dentatorubral-pallidoluysian atrophy,
EEG: Electroencephalogram
FDG-PET: Fluorodeoxyglucose positron emission tomography
HD: Huntington disease
MMSE: The mini-mental state examination
MRI: Brain magnetic resonance imaging
MSA: Multiple system atrophy
PCR: Polymerase chain reaction
SCA: Spinocerebellar ataxia
SPECT: Single photon emission computed tomography
TBP: Tata binding protein

## References

[1] Stevanin G, Brice A (2008). Spinocerebellar ataxia 17 (SCA17) and Huntington's disease-like 4 (HDL4). Cerebellum.

[2] Schöls L, Bauer P, Schmidt T, Schulte T, Riess O (2004). Autosomal dominant cerebellar ataxias: clinical features, genetics, and pathogenesis. Lancet Neurol.

[3] Rolfs A, Koeppen AH, Bauer I, Bauer P, Buhlmann S, Topka H (2003). Clinical features and neuropathology of autosomal dominant spinocerebellar ataxia (SCA17). Ann Neurol.

[4] De Michele G, Maltecca F, Carella M, Volpe G, Orio M, De Falco A (2003). Dementia, ataxia, extrapyramidal features, and epilepsy: phenotype spectrum in two Italian families with spinocerebellar ataxia type 17. Neurol Sci.

[5] Chen CM, Lee C, Chuang CL, Wang CC, Shieh GS (2010). Inferring genetic interactions via a nonlinear model and an optimization algorithm. BMC Syst Biol.

[6] Nakamura K, Jeong SY, Uchihara T, Anno M, Nagashima K, Nagashima T (2001). SCA17, a novel autosomal dominant cerebellar ataxia caused by an expanded polyglutamine in TATA-binding protein. Hum Mol Genet.

[7] Mariotti C, Alpini D, Fancellu R, Soliveri P, Grisoli M, Ravaglia S (2007). Spinocerebellar ataxia type 17 (SCA17): oculomotor phenotype and clinical characterization of 15 Italian patients. J Neurol.

[8] Maltecca F, Filla A, Castaldo I, Coppola G, Fragassi NA, Carella M (2003). Intergenerational instability and marked anticipation in SCA-17. Neurology.

[9] Nanda A, Jackson SA, Schwankhaus JD, Metzer WS (2007). Case of spinocerebellar ataxia type 17 (SCA17) associated with only 41 repeats of the TATA-binding protein (TBP) gene. Mov Disord.

[10] Nolte D, Sobanski E, Wissen A, Regula JU, Lichy C, Müller U (2010). Spinocerebellar ataxia type 17 associated with an expansion of 42 glutamine residues in TATA-box binding protein gene. J Neurol Neurosurg Psychiatry.

[11] Alibardi A, Squitieri F, Fattapposta F, Missori P, Pierelli F, Trompetto C (2014). Psychiatric onset and late chorea in a patient with 41 CAG repeats in the TATA-binding protein gene. Parkinsonism Relat Disord.

[12] Doherty KM, Warner TT, Lees AJ (2014). Late onset ataxia: MSA-C or SCA 17? A gene penetrance dilemma. Mov Disord.

[13] Park H, Jeon BS, Shin JH, Park SH (2016). A patient with 41 CAG repeats in SCA17 presenting with parkinsonism and chorea. Parkinsonism Relat Disord.

[14] Yun JY, Lee WW, Kim HJ, Kim JS, Kim JM, Kim HJ (2011). Relative contribution of SCA2, SCA3 and SCA17 in Korean patients with parkinsonism and ataxia. Parkinsonism Relat Disord.

[15] Calvini P, Rodriguez G, Inguglia F, Mignone A, Guerra UP, Nobili F (2007). The basal ganglia matching tools package for striatal uptake semi-quantification: description and validation. Eur J Nucl Med Mol Imaging.

